# Meningoencefalitis subaguda tras inmunoterapia mediante instilación intravesical con BCG

**DOI:** 10.23938/ASSN.1145

**Published:** 2026-07-03

**Authors:** Edurne Bidegain Garbala, Miren Arteaga Mazuelas, Vanesa Jarne Betrán, María Luisa Abínzano Guillén

**Affiliations:** 1 Servicio Navarro de Salud-Osasunbidea Hospital Universitario de Navarra Servicio de Medicina Interna Pamplona España; 2 Servicio Navarro de Salud-Osasunbidea Hospital García Orcoyen Servicio de Medicina Interna Estella Navarra España

**Keywords:** Meningoencefalitis, Cáncer de la Vejiga, Bacilo de Calmette-Guérin, Vacuna BCG, Bacteriemia, Meningoencephalitis, Urinary Bladder Neoplasms, Mycobacterium bovis, BCG Vaccine, Bacteremia

## Abstract

La instilación intravesical del bacilo de Calmette-Guérin (BCG), una cepa viva atenuada de *Mycobacterium bovis*, constituye el estándar de tratamiento adyuvante tras la resección transuretral del carcinoma urotelial no músculo-invasivo de riesgo intermedio y alto. La inmunoterapia intravesical produce una intensa activación inmunitaria local que ha demostrado reducir las tasas de recidiva y progresión, optimizando así el pronóstico y la calidad de vida. Aunque habitualmente es bien tolerada, pueden surgir complicaciones infecciosas e inflamatorias, tanto locales como sistémicas.

Presentamos un caso de diseminación del BCG al sistema nervioso central tras inmunoterapia intravesical, en el que la naturaleza larvada e inespecífica de las manifestaciones neurológicas iniciales dificultó el diagnóstico, retrasando el inicio de la terapia dirigida. Se resalta la importancia de revisar los antecedentes médicos y de mantener un elevado índice de sospecha diagnóstica en pacientes tratados con inmunoterapia intravesical.

## Abstract (en)

Intravesical instillation of Bacillus Calmette-Guérin (BCG), a live attenuated strain of *Mycobacterium bovis*, is the standard adjuvant treatment following transurethral resection of intermediate- and high-risk non-muscle-invasive urothelial carcinoma. Its therapeutic effect relies on intense local immune activation, reducing recurrence and progression rates and thereby improving patient outcomesand quality of life. Although generally well tolerated, both local and systemic infections and inflammatory complications may occur.

We report a case of central nervous system BCG disse-mination following intravesical BCG immunotherapy. The insidious onset and non-specific neurological manifestations complicated the diagnostic process and delayed the initiation of appropriate treatment. This case highlights the importance of thorough medical history assessment and maintaining a high index of suspicion in patients presenting neurological symptoms after intravesical BCG therapy.

## INTRODUCCIÓN

Los tumores vesicales no músculo-invasivos (TVNMI) constituyen un grupo heterogéneo de neoplasias caracterizado por una alta tasa tanto de recidiva (50-70%) como de progresión a enfermedad músculo-invasiva (10-30%) a los 5 años de seguimiento^1^. Para disminuir este riesgo, la inmunoterapia mediante instilaciones intravesicales de BCG, una cepa viva atenuada de *Mycobacterium bovis*, está indicada como tratamiento adyuvante estándar tras la resección transuretral en pacientes con riesgo intermedio y alto. Esta terapia disminuye el riesgo de recurrencia y de progresión^2^. El procedimiento es habitualmente bien tolerado; sin embargo, se describen complicaciones infecciosas en 1-5% de los casos^3^.

Se presenta el caso de un paciente con un TVNMI tratado con instilaciones intravesicales con BCG que desarrolló una infección diseminada al sistema nervioso central (SNC) en forma de meningoencefalitis subaguda/crónica. Se trata de una complicación sistémica escasamente descrita en la literatura médica científica^4^. Su curso evolutivo, caracterizado por una sintomatología vaga y un patrón clínico recidivante a lo largo de varias semanas, condicionó un notable retraso en el diagnóstico, lo que subraya la necesidad de mantener un elevado índice de sospecha en este perfil de pacientes.

## CASO CLÍNICO

Varón de 69 años con antecedentes de hipertensión arterial, hiperuricemia, diabetes mellitus tipo 2, enfermedad pulmonar obstructiva crónica, hepatopatía crónica enólica Child-Pugh A6 e hipertrofia benigna de próstata. Fue intervenido en 2015 por colecistitis aguda gangrenosa mediante colecistectomía laparoscópica. Sigue tratamiento con febuxostat, ramipril, amlodipino, metformina y dutasterida/tamsulosina. Exfumador (índice de tabaquismo acumulado de 60 paquetes-año) y bebedor de 7 UBEs/día.

En marzo de 2020 se le realizó una resección transuretral (RTU) por hematuria, con diagnostico de TVNMI grado II. En mayo de 2020, se inició tratamiento con instilaciones intravesicales semanales de 12,5 mg de BCG diluido en 50 mL de suero fisiológico. En abril de 2021 se detectó por citoscopia la primera recidiva tumoral, por lo que se realizó una RTU con hallazgo de carcinoma urotelial de bajo grado que se trató con epirrubicina intravesical semanal. En noviembre de 2021 se diagnosticó una segunda recidiva tumoral y se optó por inmunoterapia con BCG.

En enero de 2022, a las dos horas de administrar la cuarta instilación de BCG, y en probable relación a un sondaje vesical traumático, presentó un shock séptico de origen urológico sin aislamiento microbiológico que precisó ingreso en la unidad de cuidados intensivos. Se trató de forma empírica con antibioterapia de amplio espectro (piperacilina/tazobactam y amikacina a dosis estándar) con buena evolución clínica; se decidió suspender el ciclo. En mayo de 2022 presentó nueva recidiva tumoral que se trató con ciclo de inducción y mantenimiento con instilaciones de BCG, finalizando en enero de 2023.

En febrero de 2023 ingresó en Neurología por cuadro de confusión e inestabilidad en la marcha de tres días de evolución. En la exploración física destacaba apraxia, enlentecimiento psicomotor, disartria y dificultad para evocar palabras. Se realizó analítica completa con hemograma, función renal, vitaminas y perfil metabólico, con resultados dentro de la normalidad. La resonancia magnética (RM) craneal sin contraste y el electroencefalograma (EEG) tampoco mostraron alteraciones destacables. Se obtuvo líquido cefalorraquídeo (LCR) mediante punción lumbar, que mostró como hallazgos niveles aumentados de proteínas (64 mg/dL; rango normal, RN:15-45), glucosa (90 mg/dL; RN: 60-80) y células (10 hematíes/mm^3^; RN: 0; 1 leucocito/mm^3^; RN: 0-5), con adenosina desaminasa (ADA) negativo. No hubo aislamiento microbiológico en el cultivo bacteriológico del LCR. Al paciente se le administró tiamina y fue dado de alta con diagnóstico de encefalopatía de causa no aclarada en resolución.

Mostró una evolución desfavorable con empeoramiento clínico por aparición de bradipsiquia y falta de coordinación motora, por lo que a los seis días del alta acudió a otro centro hospitalario. Se repitió la RM craneal y el EEG, sin cambios. El LCR mostró persistencia de hiperproteinorraquia (94 mg/dL), glucosa 84 mg/dL y células (4/mm^3^). El cultivo bacteriológico del LCR fue negativo, así como el panel molecular FilmArray® de meningitis/encefalitis. Se solicitaron anticuerpos onconeuronales tanto en sangre como en LCR, que fueron negativos. Se completó el estudio mediante tomografía por emisión de positrones combinada con tomografía computarizada (PET/TC) que mostró un infiltrado pulmonar periférico en el lóbulo superior izquierdo (LSI) hipometabólico. En el estudio cerebral destacó el aumento de actividad en los ganglios basales y en la región temporomedial derecha, junto a hipometabolismo cortical difuso de predominio posterior. Todo ello planteó el diagnóstico de probable encefalitis límbica de origen autoinmune. Se inició tratamiento con metilprednisonlona 1 g/24 h endovenoso durante cinco días, con resolución del cuadro. El paciente fue dado de alta con tiamina y prednisona oral en pauta descendente con buena respuesta clínica al tratamiento administrado.

En mayo de 2023, dos semanas tras la realización de una instilación intravesical con BCG, el paciente ingresó en planta de medicina interna por síndrome febril de hasta 38,5 ºC sin foco claro. Se completó el estudio mediante TC toracoabdominal y ecocardiograma, sin hallazgos relevantes y sin aislamiento microbiológico en los cultivos de sangre y orina extraídos. Se administró antibioterapia de amplio espectro (meropenem y linezolid a dosis estándar) y corticoides (prednisona 10 mg en pauta descendente), con mejoría clínica progresiva. Se solicitó serología de sífilis resultando positivos los anticuerpos contra *Treponema pallidum*. Se realizó otra punción lumbar y el LCR mostró hiperproteinorraquia de 94 mg/dL, con normalización de glucosa (62 mg/dL) y células (0 hematíes y 0 leucocitos/mm^3^); ADA y VRDL (Venereal Disease Research Laboratory) negativos. El cultivo bacteriológico de LCR no detectó ningún aislamiento. Se diagnosticó de sífilis latente tardía y fue tratado con tres dosis intramusculares de penicilina benzatina 2,4 millones, administradas a intervalos de una semana. Se realizó seguimiento ambulatorio con desaparición de la fiebre, sin nueva clínica neurológica ni urológica.

**Figura 1:**
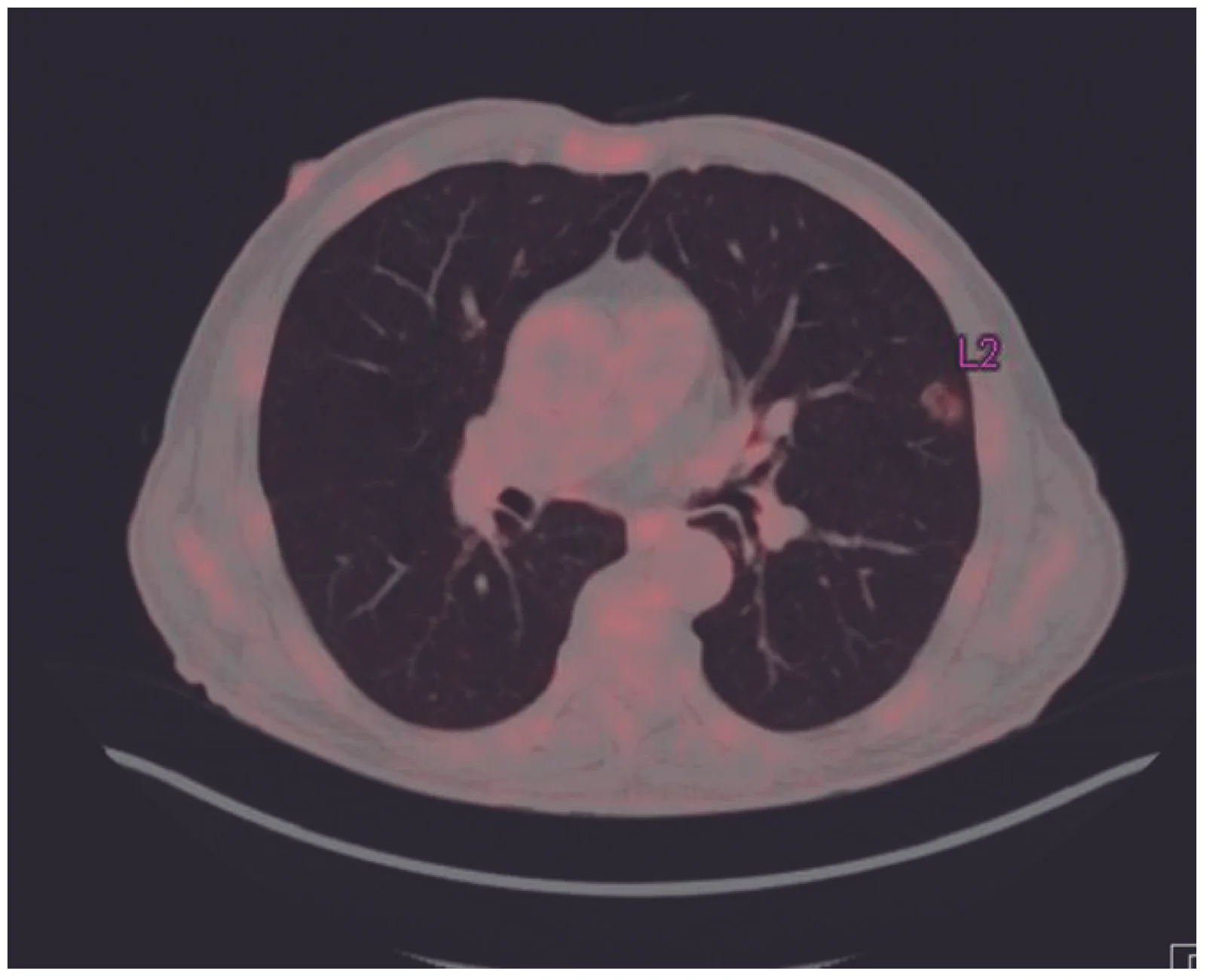
PET/TAC con F18-FDG. Leve crecimiento de un nódulo pulmonar en lóbulo superior izquierdo con patrón metabólico de baja intensidad (L2).

En octubre de 2023 ingresó de nuevo en medicina interna con síndrome febril de predominio vespertino de 2-3 semanas de evolución, sin foco claro; también presentaba astenia, debilidad y pérdida de peso. Se realizaron cultivos de sangre, orina y esputo sin aislamiento microbiológico. Se realizaron serologías con elevación de IgG para citomegalovirus, virus de Epstein-Barr, *Coxiella Burnetti*, con resultado negativo para virus de inmunodeficiencia humana (VIH) y virus de hepatitis B y C, *Brucella, C. psittasi, Borrelia, Bartonella, Francisella, Rickettsia y Leptospira*. Tanto la prueba de bacilos ácido-alcohol resistentes (BAAR) en orina y esputo, como el ensayo de liberación de interferón gamma frente a *Mycobacterium tuberculosis,* fueron negativos. Se completó el estudio con batería de autoinmunidad (ANA, ENA, ANCA, FR, antiCCP, anti-vimentina citrulinada, ECA, y anticuerpos neuronales) y marcadores tumorales (PSA, CEA, Ca 19.9, CyFRA 21.1 y Ag SCC) sin hallazgos relevantes. En la TC toracoabdominal se observó que el nódulo pulmonar en LSI descrito previamente presentaba un mayor componente sólido y un contorno más definido. Esta lesión planteó un diagnóstico diferencial que abarcaba desde naturaleza infecciosa-inflamatoria hasta metástasis, incluyendo neoplasia indolente. Además, se observaron esplenomegalia homogénea y un engrosamiento en la pared vesical anterior. La cistoscopia confirmó la recidiva del TVMNI. Se completó el estudio mediante repetición del PET/TC ambulatorio por sospecha de patología neoplásica subyacente. Este estudio mostró un discreto incremento en el tamaño del nódulo pulmonar localizado en LSI con aumento en la captación del radiofármaco aunque de baja intensidad (SUVmax=1), que incrementaba la actividad en las imágenes tardías (SUVmax= 1,3) (Fig. 1).

En noviembre 2023 se realizó seguimiento en consulta externa, persistiendo febrícula vespertina, astenia, anorexia y pérdida de 2 kg, con empeoramiento de la coordinación, temblor e inestabilidad en la marcha. No presentó cefalea ni confusión. Tras recibir el resultado del cultivo de micobacterias en orina, que mostraba aislamiento de *Mycobacterium tuberculosis* variante *bovis* BCG, se inició tratamiento con Isoniazida 300mg/día, Rifampicina 600 mg/día y Etambutol 15 mg/kg/día, además de piridoxina. Ante la sospecha de afectación del SNC, se realizó nueva punción lumbar. El LCR mostró hiperproteinorraquia 575 mg/dL (15-45), hipoglucorraquia 35 mg/dL (60-80), hematíes 3.000/mm^3^, leucocitos 300/mm^3^ con 65% mononucleares, y ADA negativo. La reacción en cadena de la polimerasa (PCR) para micobacterias en el LCR fue positivo para *Mycobacterium tuberculosis complex*. Se repitió la RM craneal, sin hallazgos, y se completó el tratamiento 8 mg/día de dexametasona en pauta descendente durante 21 días.

El paciente completó doce meses de tratamiento antituberculoso sin incidencias, con buena evolución clínica. Se suspendió de forma definitiva la inmunoterapia con BCG. El TVNMI ha continuado recidivando a pesar de recibir un ciclo de epirrubicina intravesical. En julio de 2025 fue intervenido de carcinoma urotelial de uréter medio mediante nefroureterectomía izquierda, recibiendo posteriormente quimioterapia adyuvante.

## DISCUSIÓN

La inmunoterapia intravesical con BCG constituye el pilar terapéutico estándar en el manejo de los TVNMI de riesgo intermedio y alto. Habitualmente, se inicia entre la segunda y tercera semanas posteriores a la RTU. El esquema posológico convencional comprende una fase de inducción -con instilaciones semanales consecutivas durante seis semanas- seguida de un protocolo de mantenimiento que se prolonga de uno a tres años (tres instilaciones semanales a los 3, 6, 12, 18, 24, 30 y 36 meses)^2^.

La respuesta inmunitaria desencadenada por la adhesión de BCG al urotelio vesical activa la producción de citocinas y promueve la migración local de leucocitos polimorfonucleares y macrófagos, lo que finalmente induce al sistema inmunitario local a reconocer y eliminar las células tumorales en la superficie de la vejiga^5^. Los cambios en la vejiga de los pacientes pueden mantenerse más de un año tras el primer contacto con el bacilo, aunque generalmente el efecto decrece a los 3-6 meses^5^. Algunos estudios han demostrado la persistencia de micobacterias en la orina y la vejiga de pacientes hasta 16,5 meses después de completar el tratamiento^6^. La persistencia de BCG en la vejiga puede favorecer la continuidad en la activación inmune antitumoral, pero también puede aumentar el riesgo de infecciones.

Las complicaciones asociadas con la inmunoterapia con BCG pueden ser producidas por toxicidad, infección, reacciones de hipersensibilidad y/o autoinmunidad (reactividad cruzada entre autoantígenos tisulares y antígenos micobacterianos). Las complicaciones locales son comunes y se dan en 62,8-75,2% de los pacientes. A menudo ocurren poco después de la administración de BCG y son autolimitadas a 48-72 horas. Incluyen cistitis química, cistitis bacteriana, hematuria y polaquiuria^7^. Los efectos sistémicos se dan en el 30% pacientes, siendo los más frecuentes el malestar general y la fiebre (15,5%). Mucho menos frecuentes y más graves son el rash cutáneo, la urosepsis y la infección sistémica por BCG^2^. Las manifestaciones clínicas pueden ser precoces, antes de los tres meses tras el procedimiento, como ocurre con la presentación en forma de sepsis, neumonitis o hepatitis^8^, mientras que las formas tardías pueden ocurrir mucho tiempo después de la última instilación con BCG^9^.

La infección por BCG se puede propagar desde la vejiga hacia la próstata, el pene, el epidídimo, el testículo y el riñón, produciendo infecciones locales en el tracto genitourinario. En el caso de que el bacilo acceda al tejido linfático y a la circulación sanguínea, puede alcanzar otros órganos y producir afectación sistémica (pulmonar, hepática, osteomuscular o vascular, entre otros). Estas manifestaciones a distancia pueden originarse por la diseminación hematógena inicial o bien por una reactivación tardía de la infección tras una alteración del estado inmune del huésped^8^. Se han identificado varios factores de riesgo para la diseminación hematógena de bacilos BCG durante la instilación; alteración de la barrera urotelial debido a cateterismo urinario traumático, instilación temprana después de RTU, infección del tracto urinario concomitante o disfunción inmunitaria^4^.

En este caso clínico complejo, el evento fisiopatológico inicial probablemente fue un sondaje vesical traumático durante la cuarta instilación en enero de 2022. La laceración de la barrera urotelial actuó como puerta de entrada, provocando un shock séptico inmediato y permitiendo la diseminación hematógena de *Mycobacterium bovis* hacia el pulmón, el bazo y el SNC. En febrero de 2023, la clínica neurológica con la que debutó el paciente era poco sintomática e inespecífica, y junto con la hiperproteinorraquia, se etiquetó como probable encefalitis límbica autoinmune sin cumplir completamente los criterios diagnósticos. Esta entidad comparte con el caso una evolución subaguda con alteraciones cognitivas y conductuales. Es importante mencionar que en el abordaje inicial no se descartaron de forma sistemática otras causas infecciosas, como la neurosífilis. La mejoría transitoria observada tras la administración de metilprednisolona se debió al potente efecto antiinflamatorio del fármaco sobre la respuesta granulomatosa. Este hecho enmascaró la infección subyacente y retrasó el diagnóstico definitivo, favorecido por la naturaleza paucibacilar de la afectación por *Mycobacterium bovis*.

Ante la sospecha de un proceso neoplásico, se repitió el estudio PET/TC en octubre de 2023 con un resultado indeterminado en lo que se refiere al nódulo pulmonar localizado en LSI. Se ha descrito que, mientras los valores de captación estándar máxima (SUVmax) ≤1 son altamente predictivos de nódulo benigno y los valores ≥2,5 sugieren malignidad, el PET/TC carece de especificidad para caracterizar nódulos con SUVmax entre 1,0 y 2,5^10^. El patrón de realce tardío observado concuerda con lesiones de etiología inflamatoria o granulomatosa, donde la actividad metabólica persistente condiciona una captación lenta y prolongada del radiofármaco.

Finalmente, el desarrollo de un síndrome constitucional (astenia, anorexia y pérdida de peso), la esplenomegalia, el crecimiento del nódulo pulmonar captante y el deterioro neurológico progresivo del paciente completaron el espectro clásico de una infección micobacteriana diseminada de curso subagudo-crónico. El diagnóstico definitivo se estableció en noviembre de 2023 mediante el aislamiento de *Mycobacterium tuberculosis* variante *bovis* BCG en el urocultivo, en combinación con PCR positiva para el complejo *M. tuberculosis* en el LCR, el cual ya mostraba una alteración grave compatible con meningitis tuberculosa (pleocitosis linfocitaria, hiperproteinorraquia franca e hipoglucorraquia).

La afectación del SNC por BCG es una complicación infrecuente que puede manifestarse de forma precoz o tardía^9^. Su espectro clínico incluye meningitis, tuberculomas, abscesos cerebrales, vasculitis de pequeño vaso y el síndrome de Guillain-Barré^4^. En este paciente, los síntomas clínicos, la afectación neurológica, los hallazgos analíticos y las pruebas de imagen fueron inespecíficos, dificultando su diagnóstico. Los corticoides administrados a lo largo del tiempo pudieron favorecer una reactivación de un foco latente ya que, como se ha comentado previamente, esta complicación infecciosa puede ocurrir años después de haber recibido las instilaciones intravesicales con BCG.

En conclusión, en pacientes con antecedentes de inmunoterapia intravesical con BCG, es obligatorio incluir la infección secundaria por el bacilo en el diagnóstico diferencial ante cualquier sintomatología sistémica o neurológica inespecífica. El retraso diagnóstico incrementa la morbimortalidad. Este caso subraya la necesidad de realizar una anamnesis exhaustiva de los antecedentes médicos y mantener un alto índice de sospecha clínica, especialmente ante el incremento actual en el uso de terapias inmunosupresoras o moduladoras que pueden reactivar focos infecciosos latentes.

## Data Availability

Se encuentran disponibles bajo petición a la autora de correspondencia.
